# Isolated Proximal Black Esophagus in a COVID-19 Patient

**DOI:** 10.7759/cureus.36311

**Published:** 2023-03-17

**Authors:** Nishant Aggarwal, Rabin Neupane, Unnati Bhatia, Ankur Singla, Ketan Rana

**Affiliations:** 1 Internal Medicine, Beaumont Health, Royal Oak, USA; 2 Gastroenterology and Hepatology, Beaumont Health, Royal Oak, USA; 3 Internal Medicine, Dayanand Medical College and Hospital, Ludhiana, IND

**Keywords:** histopathology examination, upper endoscopy, upper gastro-intestinal bleed, cardiac arrest, esophageal necrosis

## Abstract

Black esophagus or acute esophageal necrosis (AEN) is a rare cause of upper gastrointestinal (UGI) bleeding usually involving distal esophagus. Proximal esophageal involvement is quite rare. We present an 86-year-old female with active coronavirus disease 2019 (COVID-19) infection who came in with newly diagnosed atrial fibrillation and was started on anticoagulation. She subsequently developed a UGI bleed, which was complicated by inpatient cardiac arrest. Following resuscitation and stabilization, UGI endoscopy showed circumferential black discoloration of proximal esophagus, with distal esophageal sparing. Conservative management was instituted and fortunately, repeat UGI endoscopy two weeks later showed improvement. This describes the first case of isolated proximal AEN in a COVID-19 patient.

## Introduction

Acute esophageal necrosis (AEN) or black esophagus is a rare syndrome characterized by circumferential diffuse black appearance of esophageal mucosa with almost universal involvement of the distal esophagus, with rare cases involving proximal extension. Involvement of proximal esophagus, however, has only been limited to few case reports [1,2]. The etiology of AEN is typically multifactorial resulting from tissue ischemia, gastric reflux and loss of mucosal defenses [3]. The reported prevalence of AEN in endoscopy series ranges from 0.01 to 0.28 percent [4,5], but this is likely underreported owing to the transient nature of the injury and rapid healing [3]. Although first described by Goldenberg et al. in 1990 [6], there continues to be a lack of data regarding AEN given its rarity. We present the case of isolated proximal AEN in an elderly woman with newly diagnosed coronavirus disease 2019 (COVID-19) infection.

## Case presentation

An 86-year-old female with a recently diagnosed COVID-19 infection, was brought in via the emergency medical services for an episode of dizziness and shortness of breath. She also reported an episode of nausea and non-bloody emesis a few days ago which was deemed to be intolerance to her nirmatrelvir/ritonavir (Paxlovid^TM^). She had a known history of hypertension, prediabetes, hyperlipidemia and osteoarthritis, and her home medications were atenolol, hydrochlorothiazide, lisinopril, pravastatin, and as-needed ibuprofen. On initial evaluation, she was tachycardic (170 beats/min). Electrocardiogram showed atrial fibrillation (AFib) with rapid ventricular response (RVR). She was started on intravenous diltiazem and unfractionated heparin, which was subsequently switched to amiodarone. Two days later, she developed hematemesis, melena, and epigastric pain, accompanied by a drop in Hb from 12.8 to 10.5 g/dL. Intravenous proton pump inhibitor (PPI) therapy was initiated and she was boarded for an upper gastrointestinal (UGI) endoscopy. Her AFib continued to be uncontrolled with intermittent periods of RVR. Unfortunately, this was complicated by a cardiac arrest followed by successful resuscitation with two cycles of chest compressions and defibrillation.

**Figure 1:**
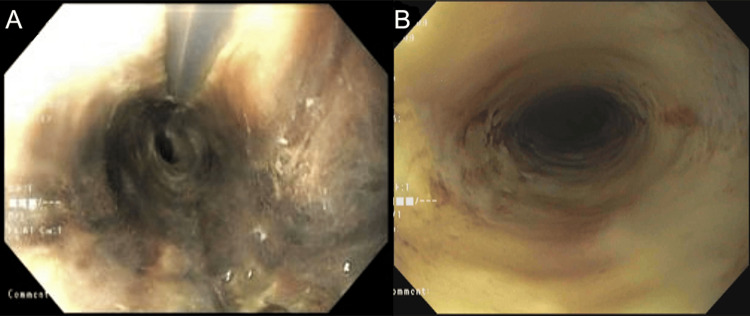
UGI endoscopy images (A) Upper gastrointestinal (UGI) endoscopy demonstrating circumferential black discoloration of the proximal esophagus. (B) Repeat UGI endoscopy demonstrating improvement in esophageal mucosa with conservative management

Two days after the resuscitation, she underwent a UGI endoscopy which showed a 4 cm long circumferential ulcer in the proximal esophagus, which was followed by a 10 cm long circumferential area with black discoloration (Figure 1A) and necrosed appearance extending from 20 cm to 30 cm from incisors. The mucosa past this segment up to the lower esophageal sphincter was normal. A 9 mm non-bleeding duodenal ulcer (Forrest class III) was also noted. Histopathology from the esophageal segment showed fibrinopurulent exudate in the absence of viable epithelium, consistent with AEN. She was initially kept NPO along with the placement of a nasoenteral feeding tube for tube feeds. Intravenous (IV) pantoprazole 40 mg twice daily was continued. No signs of perforation were noted on serial imaging. Almost two weeks later, patient underwent follow-up UGI endoscopy (Figure 1B) which showed improved appearance of mucosa and the development of a long esophageal stricture in the area of prior necrosis, which was dilated. The IV PPI was switched to oral omeprazole twice daily. Clear liquid diet was initiated, which was slowly advanced to full liquid diet at discharge. This was slowly advanced to a pureed diet with the addition of nutritive supplements and a close follow-up with a dietician for the maintenance of caloric needs. Sucralfate was added to mitigate further mucosal injury.

## Discussion

Here, we report the case of an elderly woman with a recently diagnosed COVID-19 infection followed by an in-hospital cardiac arrest, who was found to have proximal AEN on UGI endoscopy.

In a recent review by Schizas et al. (n=114), ischemic etiology was reported to be the predominant cause of AEN [7]. A “two-hit” hypothesis has been described in the pathogenesis of AEN where an initial event incites a low-flow vascular state leading to reduced regenerative capability of the mucosal barrier, which is followed by a topical injury (such as reflux of acid and pepsin). This is supported by the observation that AEN tends to occur in the distal third of the esophagus [7], which is relatively hypovascular and prone to ischemic injury. Gastric outlet obstruction can lead to increased gastric fluid accumulation, thus promoting reflux and leading to direct mucosal injury with necrosis. Similarly, acute alcohol intoxication and acute hyperglycemia can cause a transient non-obstructive gastropathy [3,8,9] thus promoting reflux, which in conjunction with the chronic detrimental effects of alcohol abuse and diabetes mellitus on the cardiovascular system, can lead to AEN. Conditions such as hiatal hernia, obesity, and post-operative state also increase the risk of reflux of gastric acid, and thus have been linked to AEN. Poor nutritional status, evidenced by accompanying hypoalbuminemia can further impair the mucosal barrier. Other causes of ischemic AEN include cardiac dysfunction, prolonged hypotension, and sepsis [7]. Apart from the above, certain infections (candidiasis, herpes simplex virus, cytomegalovirus), broad-spectrum antibiotics, severe vomiting, hypothermia, cocaine use and corrosive trauma have been linked to the development of AEN [10-12].

In our patient, the prolonged hypotension during the cardiac arrest in the setting of baseline known risk factors for microvascular insufficiency likely led to the development of AEN. AEN has been reported to be more common in the distal esophagus (97.5%, n= 79/81 [2]), but isolated proximal [1] or mid-esophageal [2] AEN with distal sparing has been noted in select few cases, as in our patient. Distal AEN has been described in patients with COVID-19 [13,14] with hypoperfusion and thromboembolic derangements secondary to SARS-CoV-2 being attributed as potential causes. The isolated proximal AEN noted in our patient may partially be the result of possible esophageal trauma during endotracheal intubation [15]. Other contributing factors may be exogenous catecholamine-induced vasoconstriction and defibrillation-induced necrosis, as has been previously described in skin, pectoralis, and myocardium [16,17].

Most patients with acute esophageal necrosis are symptomatic and present with signs and symptoms of UGI bleeding, including hematemesis and melena [3,7]. Other signs and symptoms may be related to the underlying disorder or to the development of sepsis. Accompanying laboratory findings are usually non-specific, such as anemia, leukocytosis, lactic acidosis, hyperglycemia, hypoalbuminemia, and renal insufficiency. Diagnosis is usually established by the endoscopic appearance of a circumferential black discoloration in the esophagus with friable mucosa. Although not required, histological appearance helps to confirm the diagnosis and rule out the infectious etiologies of AEN. Necrotic debris, absence of viable epithelium, and mucosal necrosis of variable depth are seen on histopathology [3]. The celiac axis serves as the common blood supply to the esophagus and the duodenum, which may explain the observed association between AEN and duodenal pathology. Other conditions which may cause the esophageal mucosa to appear dark on UGI endoscopy are melanosis, melanoma, acanthosis nigricans, and caustic ingestion, which can usually be easily differentiated from AEN by history and biopsy.

Given the rarity of the condition, limited data is available to guide management. Initial management is mainly supportive with intravenous fluids and treatment of underlying illness. Intravenous proton pump inhibitors (PPI) should be used with strict avoidance of oral intake. Submucosal epinephrine injections to treat active bleeding may have a detrimental role given the epinephrine-induced vasoconstriction can further worsen the ischemic injury [18]. Reported mortality rates vary from 13-35%, but mortality is largely due to underlying disease rather than complications of AEN [3,19]. Most commonly reported complication is esophageal stricture formation in 25-40% cases. Gurvits et al. also proposed that duodenal ulcers may reflect the severity of initial insult, and may serve as a predictor for esophageal stricture development [3], consistent with our patient. Other complications include esophageal perforation, a rare but life-threatening condition.

## Conclusions

AEN is a rare cause of esophageal ischemia presenting as UGI bleeding, especially in individuals with baseline ischemic risk factors. Even though distal AEN has been commonly reported in the literature, it may also be limited to the proximal esophagus. Management is largely supportive with strict avoidance of oral diet and PPIs. We describe the first reported case of isolated proximal AEN in a patient with COVID-19.
